# Intracerebral Abscess Caused by Actinomyces israelii

**DOI:** 10.7759/cureus.12058

**Published:** 2020-12-13

**Authors:** Dinko Stimac, Dragan Jankovic, Ljiljana Peric, Kata Anic, Christopher Nimsky

**Affiliations:** 1 Neurosurgery, Health center of Primorje - Gorski Kotar county, Rijeka, HRV; 2 Neurosurgery, University Medical Centre of the Johannes Gutenberg University of Mainz, Mainz, DEU; 3 Medicine, University of Zagreb, Zagreb, HRV; 4 Infectious Diseases, University Hospital Osijek, Osijek, HRV; 5 Neurosurgery, University Marburg, Marburg, DEU

**Keywords:** actinomyces, brain abscess, surgery

## Abstract

We describe a case of 49-years old female with a medical history of penicillin allergy, who suffered from brain infection caused by* Actinomyces israelii*. Therefore, the available therapy was metronidazole, ceftriaxone, and chloramphenicol. Due to a deterioration of the general and neurological condition of the patient, it was decided to perform a scratch skin test on penicillin, which was negative. After that, penicillin was administrated parenterally. The patient showed no hypersensitive reaction. Improvement was achieved. The patient underwent three subsequent surgeries due to primary and recurrent brain abscesses. There was a distinct improvement in her clinical status. Two months after the second re-surgery, the control computed tomography showed complete regression of the abscess.

Brain abscess caused by an *Actinomycess israelii *is very resistant to medication. However, surgical evacuation significantly accelerates the healing process. A good medication therapy is crucial and in most cases the drug of choice is penicillin. We emphasize the importance of a combined approach for treating this unusual brain infection.

## Introduction

*Actinomyces israelii* is a gram-positive bacteria within the genus *Actinomyces* which is the most frequent cause of an actinomycosis, a noncontagious, slow, purulent infection [1-3]. Actinomycotic brain infections (i.e. brain abscess, subdural and extradural empyema) are rare, but serious, life threatening infections. In a large series of pyogenic abscesses, this genus was the etiological agent in fewer than 2% of cases [2]. The present report describes a case of cranial actinomycosis in a 49-year-old female in whom no other sites of infection were found.

## Case presentation

**Figure 1:**
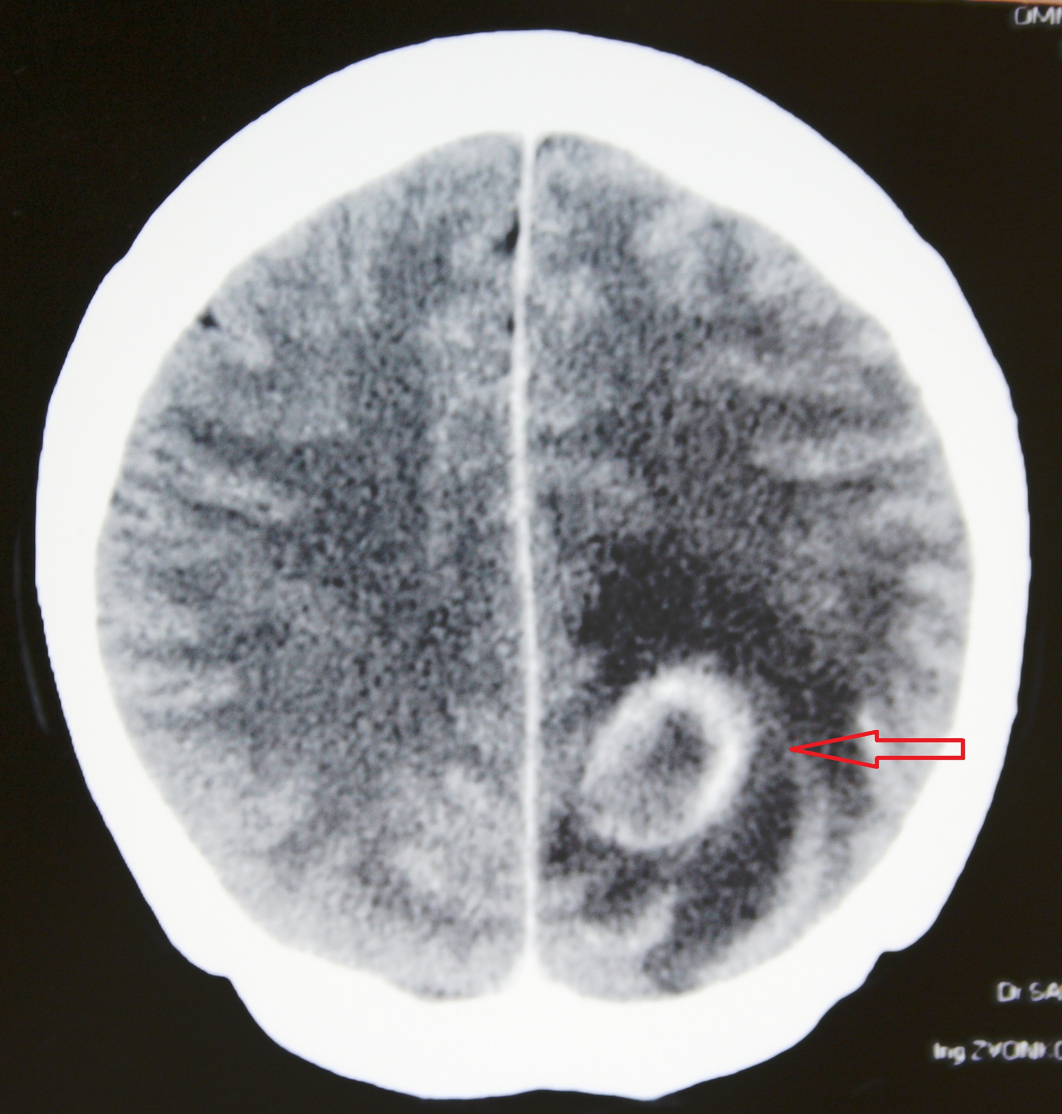
A CT scan of the brain with contrast medium obtained preoperatively revealed an expansive mass deeply in the left parieto-occipital region. A mass has the central zone of hypodensity surrounded by a ring of marked perifocal edema.

**Figure 2:**
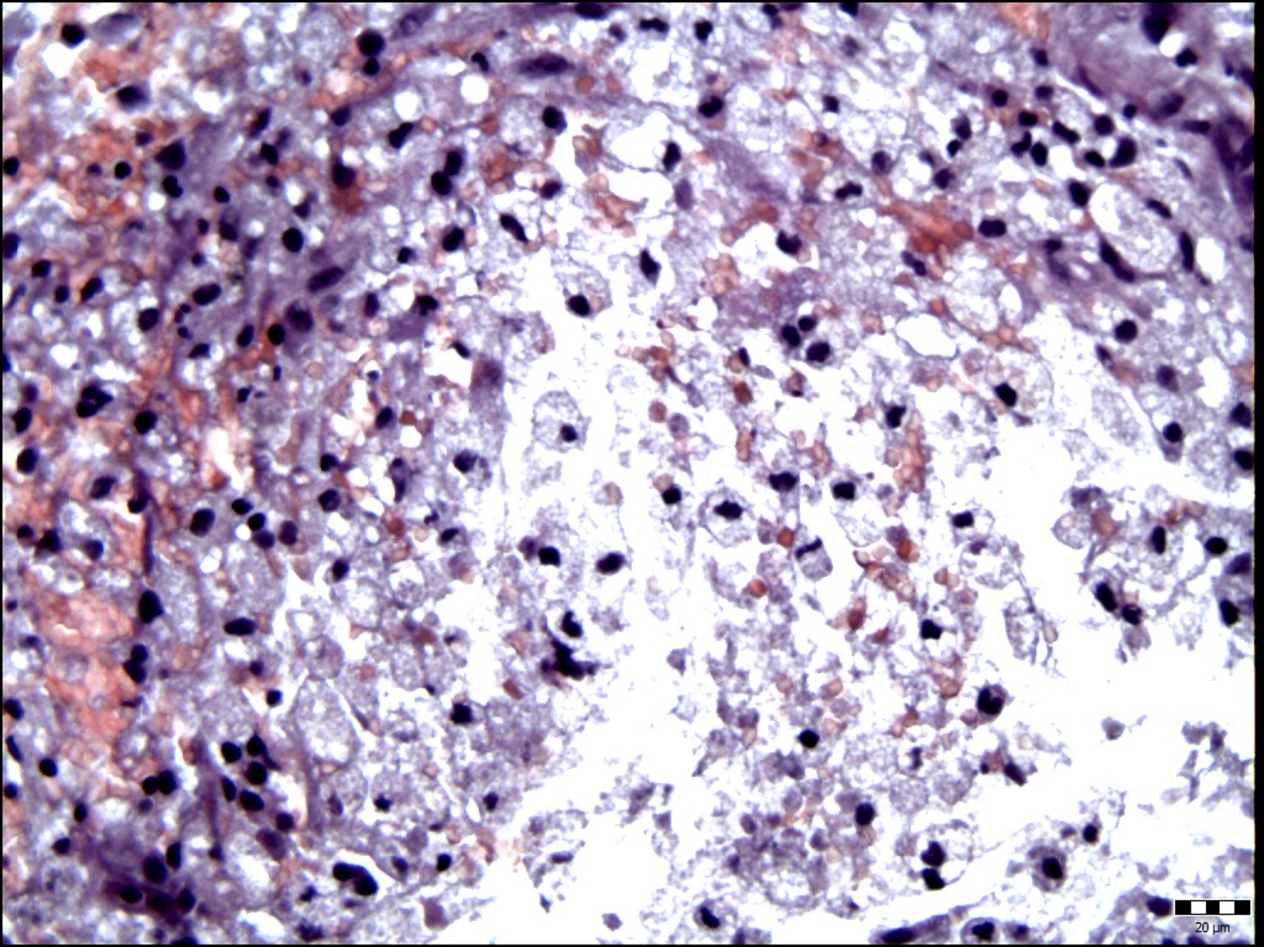
Histological examination showed brain tissue diffusely permeated with mononuclear inflammatory cells of the foamy cytoplasm with foamy macrophages and lymphocytes (hematoxylin-eosin stain, original magnification ×400).

**Figure 3:**
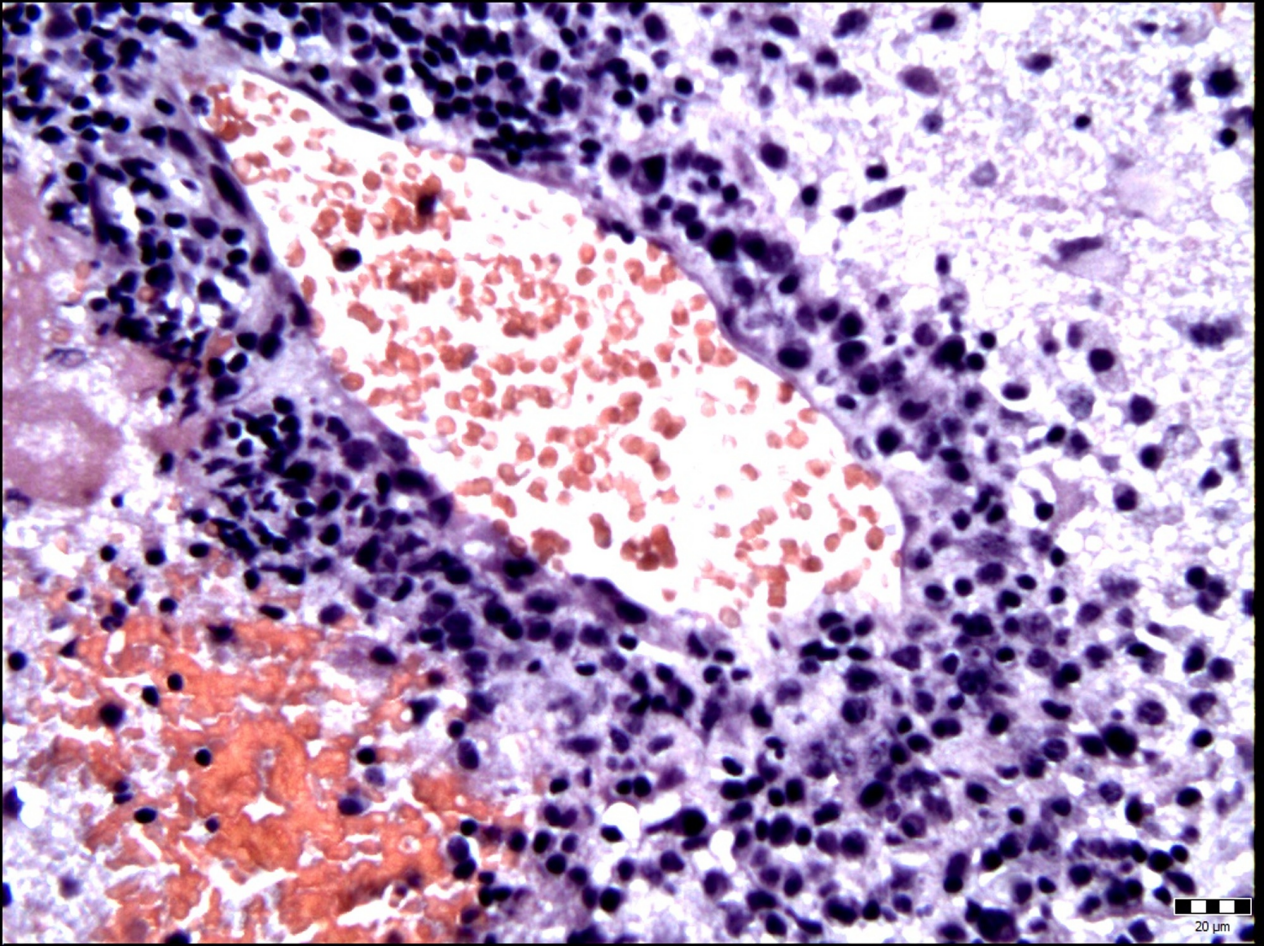
Edematous and reactively altered brain with perivascular accumulation of mononuclear inflammatory cells (hematoxylin-eosin stain, original magnification ×400).

A 49-years old female suffered from neck stiffness, paresthesia in her right leg and headache. A computed tomography (CT) scan of the brain revealed an expansive mass deeply in the left parieto-occipital region (Figure 1). The mass had a central zone of hypodensity surrounded by a ring of marked perifocal edema. The blood count results showed elevated erythrocyte sedimentation rate and leukocytosis. She had a history of good physical health and did not report any foci of infection, sinusitis, or head trauma. Two days later, her state of consciousness progressively worsened accompanied with the development of right leg paresis. Antiedematous therapy with corticosteroids was administrated and the neurological status gradually improved. The patient underwent a left sided osteoplastic parieto-occipital craniotomy six days later. An abscess with thick fibrous capsule was found intraoperativly. The capsule was opened and 15 ml of yellowish-green pus was removed. Obtained samples of pus together with small pieces of infected capsule and removed tissue were submitted for bacteriological tests. For complete abscess and capsule removal, the Cavitron Ultrasonic Surgical Aspirator (CUSA) was used. The CUSA was applied in the lowest mode of irrigation to prevent spreading of infection. The surgical procedure went well, without complications. The histopathologic examination of the excised lesion revealed the presence of *Actinomyces israelii* (Figure 2 and 3).

An antibiogram showed that the drug of choice is penicillin. The patient had previous allergic reaction to penicillin in her medical history. Therefore, according to the antibiogram, metronidazole, ceftriaxone and chloramphenicol were administrated through intravenous infusion.

**Figure 4:**
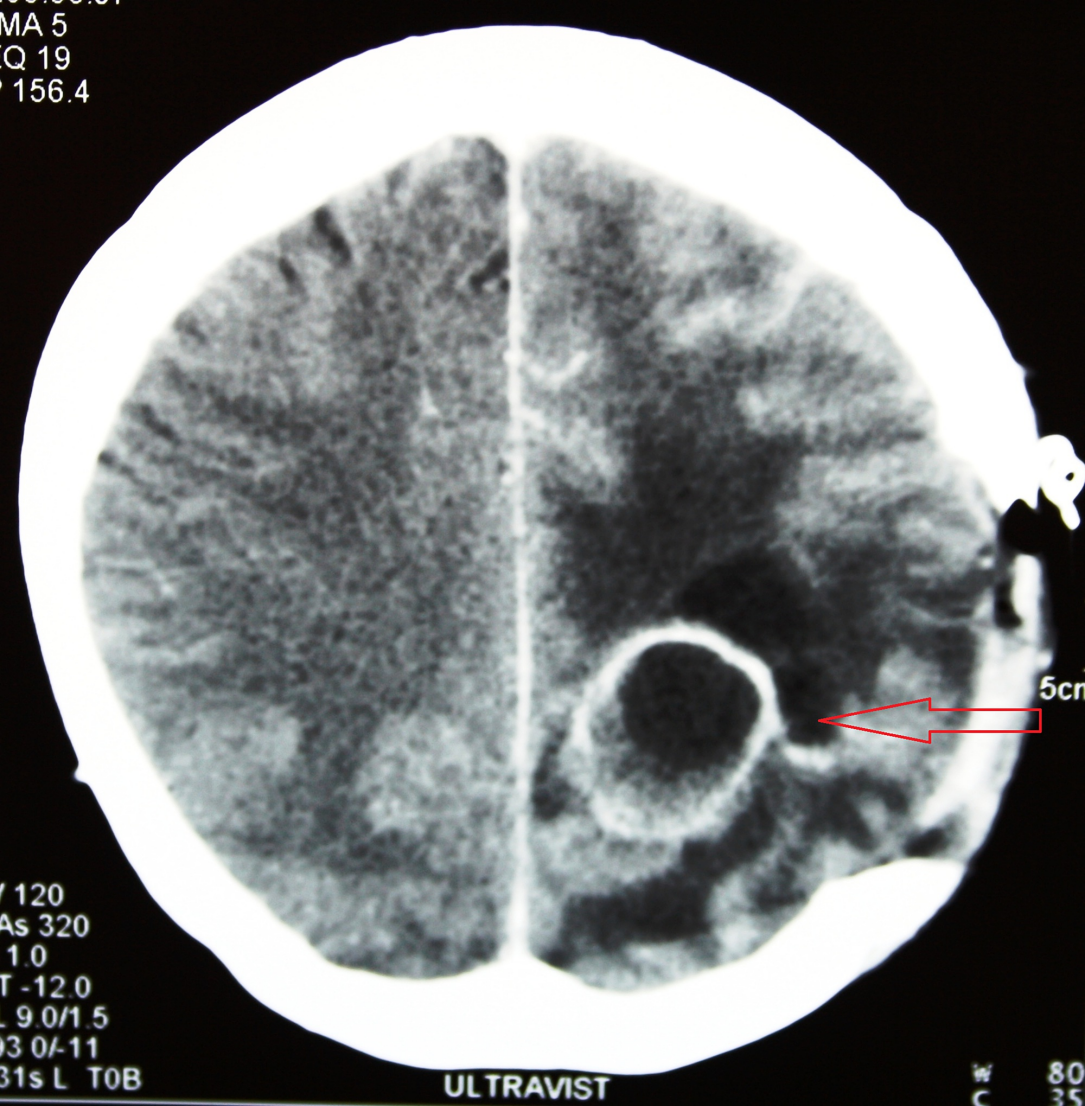
A CT scan obtained 3 weeks after first re-surgery revealed a new incapsulated process in the proximity of the one removed during the first surgical procedure.

A postoperative CT scan of the brain was performed three weeks after surgery and revealed a newly formed abscess in close proximity to the one removed before. The abscess was in its early stage of encapsulation and was close to 30 mm in diameter. A re-craniotomy was performed and the abscess was removed completely. Postoperatively, the patient´s clinical status worsened with development of sensomotoric dysphasia and alteration in conscience. Due to worsening neurological status, it was decided to perform a scratch skin test with penicillin. The test was negative and a parenteral administration of penicillin did not show an allergic reaction. Antibiotic therapy with crystacillin, ceftriax and metronidazole was continued. Rapid improvement of the clinical status was noticed. Markers for a blood infection normalized within 2 weeks. The third week after the second surgery, a control CT scan revealed another newly incapsulated process in the proximity of removed ones before, located more towards the cortex (Figure 4).

The third surgery was done and again the abscess was removed completely. The same antimicrobial therapy was continued.

**Figure 5:**
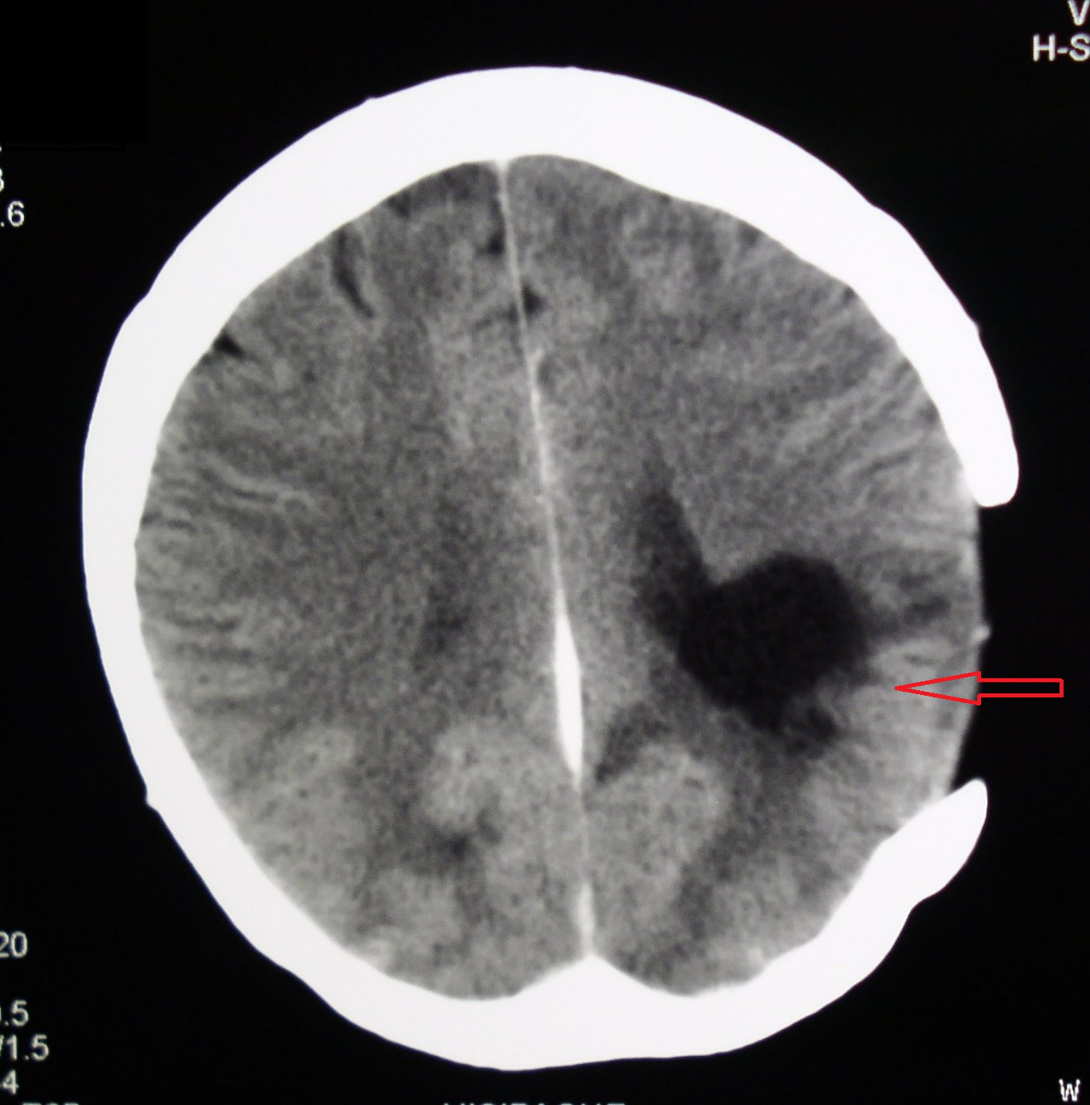
A CT scan obtained two months after the second re-surgery for the abscess. A porencephalic cyst after the abscess removal is visible. During the discharge from the hospital the patient has been without a neurological deficit.

A control CT scan of the brain obtained two months later showed complete disappearance of the abscess (Figure 5). The patient continued with antibiotic treatment for one month and was discharged fully recovered.

At the five months after surgery, the clinical follow-up examination confirmed that she had returned to her normal activities and was free of neurological deficits.

## Discussion

An *Actinomyces israelii* is a gram-positive, non-acid-fast, anaerobic or microaerophilic bacterium [4]. *Actinomyces spp.* is part of the normal commensal flora of the mouth, lower gastrointestinal tract, and female genital tract. [5]. They are very low virulent bacteria to humans because they need a mucous membrane integrity break along with the presence of devitalized tissue and other bacterial species acting as co-pathogens to cause an infection. In the pathologic state or immunodepletion, infections may occur in a local or disseminated form [6].

An intracranial actinomycosis in 45-50% cases can arise as direct extension from surrounding structures, secondary in 25% by hematogenous spread from remote site or in 10% from cranial trauma or even surgery. In 15% of all cases, the source of infection cannot be identified. Multiple brain abscesses are reported in 2-15% of cases [1].

The patients are mostly affected in the third decade of life with a male predominance [3]. Pediatric cerebral actinomycosis cases are rare. A cerebral abscess caused by* Actinomyces israelii* was detected in a 10-year-old boy with congenital heart disease and successfully treated with neurosurgical excision followed by a 4-week course of ceftriaxone [7].

The process of intracerebral abscess formation goes through four stages. The first stage (early cerebritis) is characterized with poorly demarked surrounding tissue and presence of a perivascular infiltration. In the second stage (late cerebritis), a reticular matrix and development of a necrotic center are seen. In the third stage (early incapsulation), a necrotic center with neovascularisation is observed. The fourth stage (late incapsulation) differentiates clear distinction between the collagen capsule, a necrotic center, and reactive gliosis around the capsule [8].

The patients have signs of increased intracranial pressure such as headaches, nausea, and lethargy, followed by high C-reactive protein (CRP) levels and leukocytosis over 10,000, negative hemoculture and over 90% positive lumbar puncture. Once the infection is established, spread through surrounding tissues begins with possible hematogenous dissemination at any stage of disease. In most cases of a central nervous system (CNS) involvement, it is thought to have spread from an extracranial source. When the infection has a cervico-facial localization, the principal route of entry may be the oral cavity via periapical dental granuloma [9,10,11].

In the early stages of abscess development (stages one and two) and an abscesses size of under 2.5 cm in diameter, the medical therapy is the method of choice. Antibiotic treatment is based on high doses of penicillin for a long time (6-12 months). However, Jamjoom et al. reported three cases cured by shorter antibiotic treatment [12]. The surgical intervention should be reserved for abscesses larger than 2.5 cm and in all lesions with presence of a capsule. In our case, a craniotomy was performed based on the neuroradiological findings of perifocal edema and presence of a capsule.

## Conclusions

The treatment strategy is based on the multidisciplinary approach of neurosurgeons, neuroradiologists and infectologists, while the outcome of treatment is the result of a combination of targeted antibiotic therapy and adequate surgical intervention. The choice of treatment depends on the size of the abscess, the stage of abscess formation, and the neurological status of the patient.
